# Tumultuous Atmosphere (Physical, Mental), the Main Barrier to Emergency Department Inter-Professional Communication

**DOI:** 10.5539/gjhs.v7n1p144

**Published:** 2014-08-22

**Authors:** Nasrin Jafari Varjoshani, Mohammad Ali Hosseini, Hamid Reza Khankeh, Fazlollah Ahmadi

**Affiliations:** 1 Department of Nursing, University of Social Welfare and Rehabilitation Sciences, Tehran, Iran; 2 Department of Nursing, Faculty of Medical Sciences, Tarbiat Modares University, Tehran, Iran

**Keywords:** tumultuous atmosphere, inter-professional communication, emergency department, content analysis

## Abstract

**Background:**

A highly important factor in enhancing quality of patient care and job satisfaction of health care staff is inter-professional communication. Due to the critical nature of the work environment, the large number of staff and units, and complexity of professional tasks and interventions, inter-professional communication in an emergency department is particularly and exceptionally important. Despite its importance, inter-professional communication in emergency department seems unfavorable. Thus, this study was designed to explain barriers to inter-professional communication in an emergency department.

**Methodology & Methods:**

This was a qualitative study with content analysis approach, based on interviews conducted with 26 participants selected purposively, with diversity of occupation, position, age, gender, history, and place of work. Interviews were in-depth and semi-structured, and data were analyzed using the inductive content analysis approach.

**Results:**

In total, 251 initial codes were extracted from 30 interviews (some of the participants re-interviewed) and in the reducing trend of final results, 5 categories were extracted including overcrowded emergency, stressful emergency environment, not discerning emergency conditions, ineffective management, and inefficient communication channels. Tumultuous atmosphere (physical, mental) was the common theme between categories, and was decided to be the main barrier to effective inter-professional communication.

**Conclusion:**

Tumultuous atmosphere (physical-mental) was found to be the most important barrier to inter-professional communication. This study provided a better understanding of these barriers in emergency department, often neglected in most studies. It is held that by reducing environmental turmoil (physical-mental), inter-professional communication can be improved, thereby improving patient care outcomes and personnel job satisfaction.

## 1. Introduction

An important factor that has long been recognized as the basis of quality of health care is effective communication. Effective inter-professional communication (EIC) is vital to enhancing quality and safety of care and as such a patient’s health (Scheeres et al., 2008). EIC means mutual respect toward professional values, personal abilities, use of colleagues’ knowledge and experiences, and thus seeking their opinions and consulting with them in decision making (Rostami et al., 2010). In EIC, different medical team members have common medical goals, understand each others’ roles, respect one another, resolve conflicts effectively, are flexible and use transparent communication (Sargeant et al., 2008). Many studies have shown that unfavorable inter-professional communication is an important threat to patients’ safety and health (Aiken et al., 2008; Wagner, 2011).

Because of the critical nature of work environment, the large number of staff and units, and complexity of affairs, inter-professional communication in emergency department (ED) is particularly important, and should especially be considered (Curtis & Wiseman, 2008). ED is a complex, fast-flowing department with huge amount of information exchange for providing optimal care which requires effective cooperation and interaction of the medical team (Coiera, 2008). Accordingly, due to the challenging nature of this department, in recent years, particular attention has been drawn to communication models and their application in ED (Laxmisan et al., 2007; Reddy, 2006). Establishing effective communication and accurate exchange of information in ED not only makes difference between life and death, but also induces confidence and enhances quality of treatment. For effective communication in ED, communication should be accurate, clear and transparent (Coiera et al., 2002). ED is where most medical errors occur in a hospital, compared to the hospital as a whole the ration of adverse effects estimated as between 27% and 51% compared to a ratio of between 53% and 82% in ED. A high percentage of these errors can be ascribed to ineffective inter-professional communication (Fordyce et al., 2003). It is believed that in critical situations in ED, optimal inter-professional communication can act as a key factor in reducing these errors. Given the importance of contextual role in communication and circumstances of ED on the ground, it is necessary to conduct naturalistic studies (Coiera, 2008). Other studies, while expressing concern about limited available knowledge regarding inter-professional communication, suggest that qualitative studies that help deeper understanding of factors involved should be conducted (Redfern et al., 2009a, 2009b; Patterson et al., 2013; Flowerdew et al., 2012). Since inter-professional communication varies in different social contexts, and humans’ experience of communication dependents on the incidental context, and is not separate from time, situation, or human thoughts, thus, this study aimed to identify barriers to inter-professional communication through perceptions and experiences of personnel in particular organizational, cultural and social contexts.

## 2. Methodology & Methods

### 2.1 Design

For better and deeper understanding of barriers to inter-professional communication in ED, qualitative inductive content analysis approach of Granheim and Lundman (2004) was used. Qualitative methods can provide delicate details, not easily attainable in quantitative methods (Polit, 2010).

### 2.2 Participants

Focusing on 30 in depth interviews, this qualitative study relied on a content analysis approach. To obtain different experiences, we selected participants from different medical sections and also, with maximum diversity in terms of age, gender, and work history (Table 1).

**Table 1:** characteristics of participants

Characteristic	Participants			

	Nurse	Physician	Technician	Nursing Assistant
Position(N)*	Emergency nurse (10)	emergency medicine Specialist(1)	Radiologist(2)	Nursing Assistant(1)
	Head nurse (3)	Resident(2)	Pharmacy technician(1)
	Deputy head (2)	Intern(2)	Laboratory technician(1)
	Super visors (1)			
Educational level(N)	Bachelor (15)	Medicine specialist (1)	Bachelor (1)	Diploma (1)
	MS (1)	GP (2)	MS (3)	
		Student of medicine (2)		
Age(years)	23-43	26-38	23-36	27
Gender(N)	Male(4)	Male(3)	Male(1)	Male(1)
	Female(12)	Female(2)	Female(3)	------

* Number.

Accordingly, purposive sampling was used by visiting various EDs in Zanjan City between August 2012 and July 2013.

### 2.3 Data Gathering and Analysis

Data were analyzed according to stages suggested by Granhiem and Lundman(2004) as follows: 1) Transcribing the whole interview immediately after completion, 2) reading the text to gather an overall understanding of its content, 3) determining meaning units and initial codes, 4) classifying initial codes into more comprehensive categories, and finally, 5) determining hidden content of the data. In this study, data were analyzed according to these stages, where after every interview contents were transcribed, typed and read several times to extract initial codes. The codes were combined according to similarities to form categories, and finally, hidden concepts and content of data were extracted.

### 2.4 Trustworthiness

To ensure trustworthiness of data, researchers maintained long-term engagement with the data, ensuring depth and familiarity needed to extract core themes. To ensure the codes matched experiences of participants a member check was used. Interviews, initial codes and themes were reviewed through peer debriefing, as well as external checking.

### 2.5 Ethical Considerations

This study was approved by the Ethics Committee of Tehran University of Social Welfare and Rehabilitation Sciences. Both verbal and written consent were obtained from participants. Ethical principles of anonymity, confidentiality, and right to withdraw were also observed.

## 3. Results

During analysis, 251 initial codes were extracted from a total of 30 interviews, and in the decreasing trend of final findings, 5 categories were extracted, including overcrowded emergency, stressful emergency environment, not discerning conditions in ED, ineffective management, and inefficient communication channels. From these 5 categories, tumultuous atmosphere (physical, mental) was identified as the main barrier to effective inter-professional communication. The results obtained from data analysis are presented in table 2. These areas will now be discussed in greater detail:

**Table 2:** Results from data analysis

Sub-categories	Categories	Common themes
Large number of patients		
Large number of patients’ family members	Overcrowded emergency	
Crowd as a cause of friction		
Patients’ unstable condition		
Knowledge deficiency of some doctors	Stressful emergency atmosphere	
Inconsistent presence of doctors		
Other wards’ reluctance to admit patients	Lack of appreciation of emergency conditions	Tumultuous atmosphere (physical, mental)
Personnel’s lack of appreciation of emergency conditions		
Inadequate resources		
Inconsistent supervision	Ineffective management	
Lack of proper feedback		
Defective electronic communication	Inefficient communication channels	
Defective written communication		

### 3.1 Overcrowded Emergency

It seemed evident according to the participants that overcrowded was exacerbated by presence of large numbers of patients and their family members. It is considered a very important factor in the inability to establish appropriate communication, stress, and friction between personnel. Large numbers of patients with complicated care needs create heavy workloads and fatigue for personnel, thereby reducing their tolerance and causing unintentional and inappropriate reactions, resulting in unfavorable communication, which was cited by most participants. In this respect, a participant stated: “*Busy times tire everybody and cause inappropriate communication*.” (Nurse 9, with 2-years experience). Another participant said: “*Because of contacts in a crowded emergency, too many patients, and workload, I cannot take in what the doctor says*.” (Nurse 8, with 1-year experience).

Furthermore, overcrowded emergency prevents exchange of scientific data between doctors and nurses, and thus acts as an information barrier. “…*In the crowd, I cannot find a chance to explain and transfer information*.” (Emergency medicine specialist, with 2-year experience), or “…*Staff don’t get a chance to visit their patient, and in such circumstances, no information can be exchanged between doctors and nurses*.” (Emergency supervisor, with 14-year experience).

Another consequence of large number of patients was lack of cooperation between personnel, especially between experienced and new nurses, resulting in ineffective professional relationships. In this respect, a participant argued, “*Colleagues don’t tend to help new ones, as they get really tired*.” (Interview 3, nurse 1, with 13-year experience).

Also furthermore due to overcrowded emergency and workload, principles of hygiene were not fully observed, which created difficult affects such as irritation nursing assistant. For instance: “*Nurses don’t pay attention, and drop their needle-heads on the floor, even used needles. Such cases increase at busy times*.” (Nursing assistant, with 4-year experience).

Moreover, due to busy emergency ward, when transferring patients to different wards and handing over the patient, the presence of relevant nurse was not compulsory, which led to lack of exchange of information between emergency personnel and the wards. A participant stated: “*The only ward that transfers patients without a nurse is ED and patient information is not exchanged*.” (Deputy head of a ward, with 13-year experience).

On the other hand, numerous agitated patients’ family members disrupt personnel by going to nurses and asking questions frequently exerting further pressure on personnel, contributing to display of ineffective communication on the nurses’ part. In this respect, a participant stated: “*Busy times tire everybody and cause inappropriate communication*.” Or “…*Large number of family members impedes our work, and in such situations, even in team work, communication is lost*.” (Emergency medicine specialist, with 2-year experience).

On many occasions, overcrowded emergency not only prevents establishment of inter-professional communication, but also acts as a cause of friction. In this respect, a participant said: “*Due to huge workload, nurses angrily utter ‘why don’t you transfer the patient, don’t you know how?’ This happens when work is heavy*.” (Resident 1, with 3-year experience).

3.2 Stressful Emergency Environment

It seemed evident according to the participants that stressful environment acted as a barrier to establishment of appropriate inter-professional communication, and presented as unstable patient conditions, Knowledge deficiency of some doctors, and unavailability of doctors. Unstable patient condition was a source of stress that was frequently referred to in participants’ statements. A participant stated: “*Because of unstable conditions of patients, we are constantly worried; some of us may even shout*” (Interview 1, Nurse 1, with 13-year experience).

Many participants cited stressful atmosphere of ED, and its impact on establishing appropriate communication: “*Pressure of work is huge in emergency, both mental and physical, and it is really stressful, which affects our communication*.” (Nurse 10, with 6-year experience).

Also, stress probably causes nurses not to carry out tasks on time, which leads to less communication with other personnel. *“… Emergency staffs are stressed, and fear not being able to do their work on time; everybody is busy doing her own thing, and less communicates with others*.” (Nurse 8, with 1-year experience).

Knowledge deficiency of some doctors was another theme that could contribute incorrect or belated diagnosis and treatment, thereby imposing further stress onto personnel, and acted as a barrier. “…*I have a patient that has been here three days, and they cannot diagnose what’s wrong with him, and only order consultation. This causes more stress, and communication fails*.” (Emergency supervisor, with14-year experience)

Non-continuous presence of doctor was another factor in intensifying stress in ED and ineffective communication between professions in many ways, leading to lack of decision about patients and exacerbated overcrowding in ED. A participant described the latter as follows gave these words: “*There is no attending professor, and residents don’t ring him to make a decision, they’re afraid he says what the hell they are doing here*.” (Interview 2, head of emergency department 3, with 14-year experience).

Sometimes, unavailability of doctors, and their illegible handwriting causes further stress for personnel; “*Some doctors’ handwriting is so illegible that creates more stress for us*.” (Interview 2, deputy head of emergency, with 13-year experience). Moreover, since doctors were not continually present, and alternately did their rounds, they were not closely involved in clinical duties of nurses or their care procedures, which led to ineffective communication and stress for personnel. In this respect, a participant argued: *“[The doctor] comes to visit writes such and so x-ray order, and an hour later comes in screaming the order has not been done; he is not here to see we are doing the best we can. This causes further stress*.” (Nurse 9, with 2-year experience)

Also, short presence of doctors in ED, prevented close communication between them and personnel. A nurse stated: “*As they occasionally come and give orders, and leave in a hurry, they look down on us.”* (Nurse 5, with 10-year experience), or “*We see some attending professors very little, and we shy away from them*.” (Intern 2)

### 3.3 Not Discerning Emergency Conditions

One of the reasons for ambience turmoil and subsequent ineffective communication was lack of discerning conditions in ED, which was evident at different levels throughout the hospital. “…*The supervisor is not aware that this is ED and beds should be vacant. She doesn’t cooperate with me, it gets busier all the while, and staffs are increasingly agitated*.” (Head of ED2, with 12-year experience). Other wards did not understand circumstances in ED frequently, and it displayed itself as reluctance to admit emergency patients into other wards. Often heads of other wards refused to admit emergency patients, which led to further overcrowding and agitated atmosphere in ED. “…*When I admit emergency patients, head-nurse argues with me why I don’t consider staff in that ward. She hasn’t understood emergency beds should be kept vacant*.” (Deputy Head of a ward with 13-year experience). This was also witnessed by the current researcher. Even some ED personnel did not discern emergency conditions of patients, and did not make the effort to quickly carry out procedures, which troubled other staff and led to ineffective communication. “…*You see the nurse is not bothered to carry out procedures faster for patients in bad conditions. It’s so irritating and causes friction*.” (Resident 2, with 10-year experience).

Some doctors did not understand emergency conditions either, and refused to transfer patients to wards for their own benefits, which resulted in overcrowding emergency yet again. “…*Some doctors insist that patients from ED should be on their assigned beds in wards, and this causes patients to be left in ED. When according to orders from nursing office, we transfer patients to wards and onto other beds; they would come in and make a fuss*.” (Interview 1, Deputy head of ED, with 13-year experience).

### 3.4 Ineffective Management

This was another sub-theme in ambience turmoil, which included barriers associated with inadequate resources, inconsistent supervision, and lack of proper feedback. One of the barriers associated with resources was nursing staff shortage. In this regard, a participant stated: “*Staff are continually working, with no days off, they get tired, and communication problems occur between them*.”(Emergency supervisor, with 14-year experience). Shortage of facilities and equipment comprised another communication barrier. A participant stated: “*There are a lot of stressors in ED; one is unavailability of tools, which creates conflict between nurses and doctors*” (Resident 1, with 3-year experience). Inconsistent supervision by managers led to reduced occupational motivation, resulting in lack of proper communication between personnel. In this respect, a participant stated: “*Training supervisor doesn’t come to see trainees, to see how they perform, to show what relationship they should have with a resident*.” (Interview 1, deputy head of ED, with 13-year experience). Lack of proper feedback was another barrier, “…*Whether you do or don’t do your job doesn’t make any difference*.” (Nurse 2, with 8-year experience).

### 3.5 Inefficient Communication Channels

A sub-theme of ambience turmoil was inefficient communication channels, which included deficiency in written and electronic communication. Inefficient written communication was frequently cited by participants. Doctors’ illegible handwriting caused lack of understanding doctor’s orders, waste of time for nurses, and stress. “…*Some have such a bad handwriting, and it gets worse when they are busy, causing hassle and time-wasting*.” (Nurse 7, with 3-year experience), or “*When attending professor came in the morning to read patient’s notes, resident’s handwriting was so bad, he couldn’t read it*.” (Nurse 4, with 6-year experience).

Inefficient electronic communication was another case cited by participants. This issue was sometimes as inadequate training, for instance: “*It is a new system and we have difficulty working with it*.” (Nurse 6, with 6-year experience), or, deficiency in electronic system; “*We had a lot of problems in ED, … it wouldn’t connect*.” (Intern 2). Sometimes, an ill patient needed quick action to receive medication to start treatment and it was necessary to approve it electronically. In fact, having to use electronic systems occasionally caused delays in treatment, and in vital situations, it meant excessive stress for personnel. “…*You see the patient is in a critical condition, by the time H.I.S request is sent and approval has come back, vital time is wasted, which means stress and agitation*.” (Resident 1, with 3-year experience).

Despite personnel’s knowing drug and para-clinical request procedures, sometimes, due to huge workload, this is practically impossible. “…*It was busy. They called from emergency and asked me to tick a patient’s medication [on computer] as he was being transferred to a ward. Now, I have to fill the prescription I have on the monitor first and then exit this page and do this patient’s order. Meanwhile, they ring me again, and agitate me”* (Pharmacy technician, with 6-year experience), or “*On the one hand they say, do these tests quickly, it is an emergency, and then you find H.I.S has not been done, and we don’t know what we are supposed to do*.” (Lab Technician, with 4-year experience), or “*There is no one in charge of H.I.S. Nurses do this, but they can’t when they’re busy*.” (Radiologist 2, with 10-year experience).

## 4. Discussion

This study described that overcrowding in ED was an important barrier to inter-professional communication. The descriptions are very similar to other studies have also recognized it seemed evident according to the participants that overcrowded as a barrier to effective communication (Coiera, 2008; Leape et al., 2012). Another study considered accumulation of information in busy ED as a barrier to exchanging vital information (Woloshynowych et al., 2007). There is relationship between workload and fatigue of personnel and ineffective inter-professional communication (Leonard et al., 2004). In the present study, huge workload and personnel fatigue also acted as barriers to communication according to the participants. Shortage of nursing staff in ED, consecutive compulsory busy shifts, and exhausted personnel seem to be related, in lower tolerance and agitation of personnel, which reduced their incentive to establish relationships with colleagues, reduced support and cooperation with others, and evading work. Excessive overcrowding prevented presence of emergency nurse, when handing over patients to various departments, which disrupted exchange of patient information (tests, medications, and other relevant information). Also various studies have shown, lack of face-to-face contacts when delivering emergency patients to other departments meant loss of vital information. This was considered to be an important emergency problem (Coiera, 2008; Deshkulkarni et al., 2009).

Generally, various studies have shown that overcrowded workplace imposes pressure and additional fatigue on personnel, and through reducing threshold of irritability, may leads to unfavorable behaviors or communication that fails to support the high level of technical skill needed, and prevention of exchanging vital information. Meanwhile, Azimi et al. (2010) considered critical conditions in ED the reason for closer cooperation of medical team and better communication. It seems if personnel are suitably trained to deal with crisis in emergency, the atmosphere is generally unemotional. During busy times, since medical team members mutually need cooperation with one another, the atmosphere is maintained, and may even get effective partnering. However, when the atmosphere is emotional, close communication cannot take shape, and busy ED intensifies interrupted communication, as was seen in the present study.

In the present study, stressful atmosphere in ED acted as a barrier to establishing proper inter-professional communication. Instability in ED, patients’ conditions, over-admission of patients beyond ED capacity added to already stressful atmosphere in this department. This finding is similar to that found in a study, where workplace stress was proposed as a barrier to inter-professional communication (Leonard et al., 2004) and overcrowding was considered a stressing factor in ED and a possible contributor, according to the current participants, of ineffective communication (Leape et al., 2007) and ineffective communication as the contributor of occupational stress (Rostami et al., 2010). Thus, it can be inferred that a vicious cycle is created here, and workplace stress prevents formation of proper inter-professional communication, which in turn intensifies stress in the workplace. Generally, stressful atmosphere of ED is due to its challenging, fast pace, busy nature, and negative impact of this stress on communication has been identified in many studies. Thus, to improve inter-professional communication, factors that intensify stress should be reduced from ED.

In the present study, knowledge deficiency of some physicians was identified as a communication barrier by intensifying stress in the emergency. Unnecessary and irrelevant admissions and lack of quick decisions about patients exacerbated stressful atmosphere of ED, all resulted from knowledge deficiency of some physicians. Shannon et al. (2012) stated wrong or delayed diagnosis was the most common reason for dysfunctional ED. Nugus et al. (2011) found that high levels of knowledge and skills of emergency doctors effective in creating calmer ED atmosphere. In the present study, another consequence of doctor’s deficiency of knowledge and skills frequent orders and changing orders by residents, which acted as a big barrier to communication. This was in line with results by Hargestam et al. (2013) that found a large number of orders lead to over-loading responsibilities of resuscitation team, and had negative impact on the function of medical team members and their relationship. Also, Jobbour et al. (2013) found that the application of specific treatment protocols in the emergency room contribute to improve quality of care and inter-professional communication.

In the present study, inconsistent presence and unavailability of doctors was another cause of stress and acted as another communication barrier. Also, hasty attendance of doctors in ED prevented formation of close relationships with personnel. Gotlib et al. (2012) found that continuous and reliable presence and availability of doctors led to establishing informal and friendly relationships with nurses, and resulted in better inter-professional communication. Cresvick et al. (2009) also found availability of doctors reinforced a sense of belonging to a bigger group in personnel, and reduced the feelings of being alone. Tjia et al. (2009) also considered unavailability of doctors and long wait for telephone contacts a major barrier to communication. Generally, it seems, physical presence of doctors alongside other personnel created a sense of support in them, and was effective in creating friendly relationships. However, in the present study, in addition to this issue, inconsistent presence of doctors prevented communication that could enhance inter prof contact and communication in other ways as well. On the one hand, it meant no decision for patients, and intensified overcrowding in ED, which together with huge workload and fatigue, prevented proper inter-professional communication. Furthermore, since doctors were not in attendance continually, and visited wards alternately, they could not closely oversee nurses’ clinical work or their care procedures, and this led to ineffective communication. Shannon et al. (2012) also found that physical distance between doctors caused broadening of communication gap between them. In the present study, both physical distance and hierarchical culture (mental distance) between doctors and medical students caused failure to establish proper communication between them. There was both physical and mental distance, and fear of the superior caused medical students not to comfortably discuss medical issues with them. Therefore continuous presence of experienced doctors is among requirements in ED.

In the present study, lack of understanding emergency department conditions was considered another barrier to communication. However, in review of literature, no study was found on this issue, which may be due to special attention to ED and specific training courses on importance of ED and its requirements in other studies; a void that is deeply felt in the present study.

In the present study, ineffective management was another barrier to inter-professional communication, which included barriers associated with lack of resources, disjointed supervision, and lack of feedback. These results are in agreement with Azimi et al. (2010) and Spenser et al. (2002) studies. However, the present study results disagree with results of a study by Keshtkaran et al. (2012) in which, in staff’s opinion, most managers enjoyed good communicational skills, and thus were able to establish effective communication among staff. They identified effective verbal, listening, and feedback skills as essential components in managers’ communicational skills. Generally, ineffective management, through lacking managerial and communicational skills, can act as a major barrier to inter-professional communication. Thus, to strengthen communication, utilizing effective and efficient management is essential.

In the present study, inefficient communication channel, in written and electronic forms was an issue. Redfern et al. (2009a) also identified high levels of communication failure in written form, only, in the present study, this issue existed in ED, and doctors’ bad handwriting was an impediment to maintaining written communication. But, Redfern et al. (2009b) considered transfer of written information from ambulance to ED as a defect in written communication. Accordingly, attention to written communication and elimination of existing barriers, are considered among priorities of ED. It seems essential to impose appropriate policies and warning doctors to write their orders in a more legible handwriting. Moreover, in the present study, problems existed in use of electronic devices and occasionally the system itself, which prevented establishment of timely and speedy communication. Coiera et al. (2008) also identified technical problems as a disruptive factor in ED communication. Proper training of personnel, with respect to use of these communication facilities and eliminating technical problems are necessary in establishing fast communication in ED, where speed is the first and the most essential issue.

## 5. Conclusion

In this study, principle barriers to establishing inter-professional communication in ED were identified. However, distinguishing barriers in practice is not easily possible. In fact, it can be argued that barriers associated with tumultuous atmosphere in emergency department create a chain of major limitations in establishing desirable inter-professional communication, and identifying main inhibiting factors can be effective in removal of other barriers. Accordingly, reduction of stress and factors that exacerbate stress in ED for establishing appropriate communication is essential. Emergency nurses can increase their knowledge and skills for promoting inter-professional communication. Because the present study results were obtained through real experiences and perceptions of participants in the field, they can be used in designing appropriate interventions to enhance inter-professional communication. The present qualitative study is indicative of experiences and perspectives of different medical professions within Iranian context and culture. Therefore, the transferability of findings should be considered with caution and critiqued and compared with those of similar studies conducted in other contexts.
